# Diverse Bacterial Anti-Phage Strategies: From the Laboratory to the Clinic

**DOI:** 10.3390/cimb48020191

**Published:** 2026-02-08

**Authors:** Yong Shao, Zhu Gao, Ying Zhang, Jianqiong Zhang

**Affiliations:** 1 School of Life Science and Technology, Southeast University, Nanjing 211189, China; 230228997@seu.edu.cn (Y.S.); 220233922@seu.edu.cn (Z.G.); 2 Department of Microbiology and Immunology, School of Medicine, Southeast University, Nanjing 211189, China; 3 Jiangsu Provincial Key Laboratory of Critical Care Medicine, Medical School, Southeast University, Nanjing 211189, China

**Keywords:** phage therapy, phage resistance, bacteria–phage interaction, anti-phage defense, multidrug-resistant bacteria

## Abstract

Refractory infections caused by multidrug-resistant bacteria have emerged as a substantial threat to public health, prompting renewed interest in phage therapy. Bacteria and phages are ubiquitous in diverse environments, engaging in continuous interaction and co-evolution. In response to phage infection, bacteria have developed an array of defense mechanisms. Current studies on bacteria–phage interactions predominantly focus on laboratory settings using artificial media, whereas the final goal of phage therapy—to combat antibiotic-resistant bacteria—lies in its clinical application. This review describes bacterial defense strategies against phage infection in the context of laboratory-based artificial media, animal experiments and clinical cases, aiming to deepen the understanding of bacteria–phage interactions and promote the advancement of effective phage therapy for clinical applications.

## 1. Introduction

Bacterial infections, particularly those caused by multidrug-resistant bacteria, pose a significant threat to human health and safety. According to projections, the global annual mortality attributable to antimicrobial resistance is anticipated to reach 8.22 million by 2050 [1]. Bacteriophages, viruses that specifically infect bacteria, offer several therapeutic advantages: strict host specificity without disrupting the normal flora [2,3]; self-replication without repeated administrations [3]; biofilm degradation, increasing bacterial susceptibility to antibiotics and promoting potential phage–antibiotic synergy [2,4]; and synergistic interaction with the host’s immune system to promote bacterial clearance [2,5]. Phage therapy is emerging as a viable therapeutic alternative to conventional antibiotic treatment. However, a narrow host range and phage resistance have constrained the rapid advancement of phage therapy.

To overcome the limitation of a narrow host range, the prevailing strategy involves using phage cocktails, which have exhibited improved efficacy. Nevertheless, there remains a lack of effective solutions to phage resistance (i.e., bacteria developing resistance to phages they were previously sensitive to). A major contributing factor is that evolutionary adaptability enables bacteria to continuously evolve to withstand phage infection. Elucidating the mechanisms underlying phage resistance holds promise for uncovering new avenues in phage therapy and potentially advancing next-generation therapeutic strategies for addressing clinically refractory bacterial infections.

Prior studies have predominantly concentrated on in vitro experiments and have made remarkable progress [2,3,4,5]. In this review, we synthesize the latest findings regarding phage resistance in both in vitro and in vivo settings and provide a detailed discussion of the resistance mechanisms observed in clinical phage therapy and animal experiments. The primary objective is to provide some support for understanding bacteria–phage interactions and for facilitating the clinical translation of phage therapy.

## 2. Emergence and Mechanisms of Phage Resistance Under Laboratory Culture Conditions

### 2.1. Inhibiting Phage Adsorption

**Figure 1:**
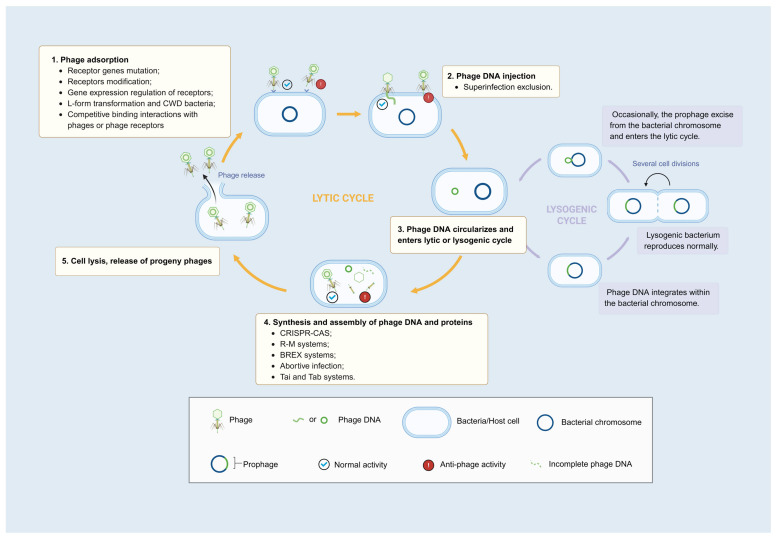
Phage life cycle and bacterial anti-phage defense strategies at each stage. Lytic phages, commonly employed in bacterial infection control, predominantly follow the lytic cycle (left circle). In contrast, temperate phages typically integrate their genetic material into the host genome upon infection, a process termed the lysogenic cycle (right circle). The documented anti-phage defense strategies primarily operate during the lytic cycle (defense systems that function at specific stages are listed in the box). Created in BioRender. Shao, Y. (2026) https://BioRender.com/8401770 (accessed on 4 February 2026).

The initial step in the phage life cycle is the adsorption of host bacteria, which requires a specific receptor for successful attachment [6,7,8]. This ligand–receptor interaction underpins the high specificity of phages. To evade phage infection, many bacteria employ countermeasures at the initial stage of infection by reducing phage receptor availability, thereby hindering or preventing the original phage from attaching to the bacterial surface and, consequently, blocking the infection process (Figure 1).

#### 2.1.1. Phage Receptor Gene Mutations

Many bacteria influence phage adsorption by mutating receptor genes. These resistant strains may also arise as a result of selective pressure; specifically, strains with only receptor mutations are incapable of lysis during phage infection and ultimately survive and proliferate. Common means of receptor gene mutation include (i) SNP (single-nucleotide polymorphism), where a single base can lead to receptor-coding gene mutation; (ii) transposition, leading to truncation or elongation of ORFs encoding receptors, produces incomplete or non-functional receptors.

Fang et al. [9] demonstrated that the *K. pneumoniae* strain B0 exhibited a rapid morphological transition from mucoid to dry colonies upon exposure to phage P24. Through the sequencing of resistant mutants, mutations in two genes critical for capsule biosynthesis were identified: a frameshift mutation in *mshA* caused by an SNP and an insertion in *wcaJ* encoding glycosyltransferase. The phage adsorption rate to mutant strains significantly decreased, indicating that mutations in these genes effectively prevented phage P24 adsorption and conferred phage resistance.

#### 2.1.2. Masking or Modification of Phage Receptors

Beyond receptor mutation, certain bacterial species employ alternative strategies to impede phage adsorption and subsequent infection, primarily through receptor masking or structural modification. This approach effectively alters the surface recognition sites targeted by phages [10,11,12,13,14,15,16]. Recently, Gaborieau et al. [10] reported that upon infection by phage 536_P1, two distinct types of phage-resistant strains emerged in *E. coli* 536: one involves receptor deletion, and the other involves receptor masking. The former is characterized by at least one mutation in the gene involved in LPS (serving as the phage receptor) biosynthesis, preventing phage adsorption. The latter is marked by a mutation in the capsular coding region, leading to a thicker capsule in mutant clones that obstructs phage adsorption to the receptor. In addition, in vivo virulence assays were conducted, revealing that the absence of the LPS significantly impaired bacterial virulence. Conversely, the resistance mechanism that masked the receptor preserved virulence due to the presence of functional LPS. These findings suggest that the fitness trade-off imposed by phage resistance does not invariably result in reduced bacterial fitness and underscore the necessity for a deeper understanding of phage resistance to develop strategies for clinical therapy. In addition, for certain phages, receptor modification (such as glycosylation) is a prerequisite for their successful adsorption [11,12,13,14,15,16]. For these phages, the loss of a glycosyl may lead to the development of phage resistance.

#### 2.1.3. Regulation of Phage Receptor Gene Expression

Phage receptors are encoded by unique genes, and their expression is theoretically regulated through complex and precise mechanisms. Høylden-Kroghsbo et al. demonstrated that *E. coli* can decrease the expression level of the λ receptor LamB via AHL quorum-sensing (QS) signaling, leading to a substantially reduced phage adsorption rate and, consequently, enhancing the survival of the bacterial population [17].

In contrast, QS can also enhance receptor expression and facilitate phage infection. *V. alginolyticus* employs QS to upregulate ugd expression, which results in increased synthesis of capsular polysaccharides (CPSs) serving as phage receptors, thereby promoting increased susceptibility to phage infection [18]. Notably, the use of a QS-signaling inhibitor led to a reduction in type IV pili (T4P) expression, decreasing phage adsorption [19]. These results suggest that QS can be artificially modulated to interfere with phage–bacteria interactions.

#### 2.1.4. L-Form Transformation and CWD Bacteria

Many phages infect bacteria through bacterial cell walls. However, this process can be evaded by shedding of the cell walls, which is known as “L-form transformation” [20,21]. Wohlfarth et al. [20] demonstrated that *L. monocytogenes* is capable of evading phage predation by transitioning into an endolysin-mediated L-form state. However, the cell-wall-free state is not permanent. L-form bacteria can rapidly revert to a walled state in the absence of a phage, enabling them to effectively address other impending survival pressures [20].

Similarly, Veronique Ongenae et al. [21] reported that the *Streptomyces* strain MBT86 transiently loses its cell wall following phage exposure, generating viable cell-wall-deficient (CWD) cells. This adaptation enhances the population’s survival rate. In addition, artificially induced CWD cells can also withstand phage infection. Notably, CWD cells were unable to proliferate in contrast with L-forms but would switch back to a walled growth mode under certain conditions [21].

#### 2.1.5. Competitive Binding Interactions with Phages or Phage Receptors

The interaction between phages and receptors is subject to modulation by specific bacterial-derived substances. These substances can competitively inhibit the phage–receptor interaction through either phage binding or receptor occupation. For example, microcin J25 secreted by bacteria can specifically bind to FhuA, which acts as the receptor of phages T1 and T5, and competitively inhibits phage adsorption [22]. In addition, research indicates that phages can induce the release of bacterial outer-membrane vesicles (OMVs). Because of sharing certain identical components with the bacterial outer membrane, OMVs can specifically bind to phages, affecting phage adsorption [23,24,25,26].

### 2.2. Interfering Cell Entry

If bacteria fail to block phage adsorption, the next step is “intrusion”, where the phage attaches to the host cell and injects genetic materials [27]. Here, bacteria primarily use the superinfection exclusion (SIE) system to prevent phage infection (Figure 1). SIE systems, mediated by prophage-encoded proteins, function at the cell surface by either (i) preventing phages from binding to receptors [28,29,30,31] or (ii) inhibiting the injection of their genomes into the bacterial envelope [27,32,33,34].

It has been demonstrated that T4P serves as the receptor necessary for many phages to infect *P. aeruginosa*, and mutations in T4P biosynthesis-related genes can result in phage resistance [35,36,37]. Furthermore, numerous phages encode proteins that suppress T4P function to prevent superinfection. For instance, Tip, a protein encoded by phage D3112, interacts and inhibits PilB activity, which is essential for T4P biosynthesis. This interaction leads to the inability of subsequent phages to infect via the receptor T4P [28]. Similarly, the Aqs1 protein from phage DMS3 suppresses T4P by binding to PilB [31]. Moreover, the filamentous phage protein PfsE interacts with PilC to block T4P, causing superinfection exclusion [29].

In addition to the encoded phage receptor repressor protein, another sophisticated approach entails thwarting the injection of phage genetic material. For example, SieA, a protein encoded by phage P22, wards off superinfection by P22-like phages through the inhibition of phage DNA translocation across the bacterial inner membrane [27]. Likewise, Gp15, a product of the E. coli phage HK97, obstructs the DNA injection of both HK97 and HK75 via its intricate interaction with the TMP-PtsG complex [33,34].

### 2.3. Disruption of Phage DNA and Protein Synthesis and Assembly

#### 2.3.1. CRISPR-CAS Systems

Is adaptive immunity unique to eukaryotes? The answer is negative. In 2007, Barrangou et al. proved the role of CRISPR-Cas as an adaptive immune system in prokaryotic organisms [38]. Its action mechanism can be divided into three distinct stages [39]: (i) adaptation; (ii) expression and maturation; and (iii) interference. In the first phase, host Cas proteins recognize foreign target DNA, acquire new spacer sequences, and integrate these sequences into CRISPR arrays to establish an adaptive immune memory. In the subsequent phase, CRISPR arrays are transcribed into pre-crRNA transcripts, which are subsequently processed into mature crRNAs. These crRNAs bind to one or more Cas proteins, forming functional Cas-crRNA complexes. In the final phase, the Cas-crRNA complex identifies foreign nucleic acid targets via base pairing with the crRNA complementary sequence. Upon successful recognition, the complex catalyzes the cleavage and degradation of the target nucleic acid. CRISPR-Cas systems have been extensively described in a previous report [39]. Herein, we only present a succinct overview.

#### 2.3.2. Restriction Modification Systems

Restriction modification (RM) systems protect the host’s genome by epigenetically modifying it, thereby distinguishing it from unmodified foreign DNA, which is subsequently destroyed. Most RM systems comprise two components: DNA methyltransferase (MT) and a restriction endonuclease (RE) that recognizes and cleaves unmethylated DNA [40]. Through methylation of their own nucleic acids, bacteria can protect their genomes from self-cleavage. Notably, the type IV RM system differs from other types in that it specifically targets and cleaves DNA sequences containing specific modifications. With in-depth research, several restriction modification systems have emerged in recent years, including the SspABCD [41,42], qatABCD [43], PD-T7-1 [44] and DISARM (Defense Island System Associated with Restriction Modification) systems, providing a broad range of protection [45].

#### 2.3.3. Bacteriophage Exclusion Systems

Bacteriophage exclusion (BREX) systems are identified in ~7% of prokaryotic genomes [46,47], representing a markedly lower prevalence compared with the ~83% observed for RM systems [47]. The principle of BREX action is similar to that of the RM system, both involving the methylation of the host bacterial genome DNA [46,47,48]. The difference lies in the fact that the RM systems use RE to recognize and degrade unmethylated phage DNA, whereas the BREX systems inhibit the replication of foreign DNA after recognition without immediately cutting it [48]. In addition, the BREX systems typically use BrxX (PglX) for methylation, which are more sophisticated than the RM systems [48].

#### 2.3.4. Abortive Infection

Abortive infection (Abi) inhibits phage proliferation by triggering bacterial cell suicide or growth arrest [49]. Broadly, Abi is not a defense system, but rather an immune strategy that manifests in various defense systems encoded by bacteria [49]. Each Abi system comprises at least two functional modules: one responsible for detecting phage infection and the other for killing bacteria or shutting down bacterial metabolism post-detection. The latter module must be strictly regulated to avoid interfering with normal bacterial growth [49,50]. The early-discovered and most extensively studied Abi systems include Rex and abiZ, among others. The Rex system is composed of RexA and RexB, in which RexA can sense protein–DNA complexes from phage replication or recombination and then activate RexB. Activated RexB forms ion channels in the bacterial inner membrane, causing membrane potential loss and intracellular ATP reduction [51,52]. AbiZ works synergistically with endolysin and holin to expedite bacterial lysis, releasing incomplete and non-infectious phage particles [53].

In addition, the toxin within toxin–antitoxin (TA) systems has the ability to inhibit bacterial growth and induce cell death. The general action mechanisms can be summarized as follows: (i) DNA cleavage, disrupting DNA replication, as observed with RalR [54]; (ii) RNA cleavage and translation inhibition, as exemplified by MazF [55]; and (iii) damage to cellular structures, as demonstrated by DinQ [56]. Recent research found that the TA system can respond to the perception of flagella during phage DNA injection, activating bacterial defense [57].

**Figure 2:**
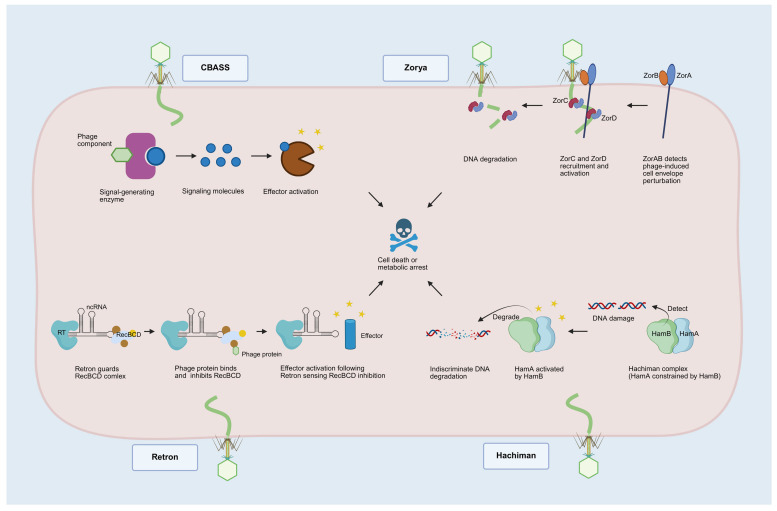
Simplified schematic diagram of the four representative systems of Abi. Arranged sequentially from left to right and top to bottom, they are CBASS, Zorya, Retron, and Hachiman systems, respectively. While the mechanisms through which different systems induce Abi vary, they converge on a common outcome: the cessation of bacterial cell death or metabolic arrest. Created in BioRender. Shao, Y. (2026) https://BioRender.com/p3hmfj2 (accessed on 4 February 2026).

Cyclic oligonucleotide-based anti-phage signaling systems (CBASSs; Figure 2) represent a large family of Abi systems identified in recent years. Upon recognition of phage infection, a cyclic dinucleotide or trinucleotide second messenger is generated, activating proteins responsible for Abi-mediated cell death [58,59]. Cohen et al. confirmed that phage infection induces cGAMP synthesis, activating phospholipases that disrupt membrane integrity and cause host cell death before phage proliferation completes [58]. In addition to cGAMP, CBASS systems utilize a variety of cyclic oligonucleotide signals, such as cyclic AMP-UMP, cyclic UMP-UMP, cyclic AMP-AMP-GMP, and others [59]. Beyond these classical nucleotides, dITP can also function as a signaling molecule [60], highlighting the importance of non-classical nucleotides in bacterial immunity.

Retron functions as an anti-phage defense system comprising reverse transcriptase (RT), non-coding RNA (ncRNA) and effector proteins (Figure 2) [61,62,63,64]. Millman et al. [61] found that retron Ec48 activates its toxicity upon phage-induced RecBCD inhibition [65], causing abortion and subsequent cell death. Another well-studied retron system is Ec86. Phage-encoded DNA cytosine methyltransferase (Dcm) is a trigger for the EC86 system, which is activated following msDNA (multi-copy single-stranded DNA) methylation, leading to NAD^+^ hydrolysis and resulting in abortive infection [62,66].

The Hachiman system is a heterodimeric nuclease–helicase complex, where HamA is the effector nuclease, and HamB is the sensor helicase (Figure 2) [67]. Normally, HamB restricts HamA’s activity when the host DNA is intact. However, upon detecting DNA damage, HamB activates HamA, triggering DNA degradation within the cell, including both phage-derived and host-derived DNA, and forming “ghost” cells devoid of DNA [67]. The Hachiman system appears to combat phage infection in a manner analogous to TA systems. However, the interaction between HamA and HamB is not antagonistic, as HamB functions both to inhibit and activate HamA, which deviates from the typical characteristics of TA systems.

Zorya, a newly discovered bacterial immune system, comprises two core components, ZorA and ZorB, and additional functional proteins (Figure 2). For instance, type I Zorya includes ZorC and ZorD, with ZorD exhibiting helicase activity [68,69]. ZorAB is activated in response to phage invasion and subsequently transmits invasion signals to recruit and activate the effector proteins ZorC and ZorD, thereby facilitating phage DNA degradation [69]. Type II Zorya lacks *zorC* and *zorD* but contains *zorE* encoding HNH endonuclease [43,66] for T7 phage defense [43]. In addition to phage genetic material, Zorya-mediated phage defense encompasses the death or metabolic arrest of infected cells, thus categorizing these events as abortive infections.

Numerous systems are associated with Abi, and in recent years, other systems conferring phage resistance through NAD^+^ consumption have been identified, including SIR2 [63,70,71,72], Thoeris [73] and DSR2 [74], which will not be discussed in detail here.

#### 2.3.5. Tai and Tab Systems Influencing Phage Tail Assembly

The tail assembly inhibition (Tai) system protects the bacterial population by preventing the assembly of phage tails and making the newly synthesized phages lose their tails to infect new host cells. Unlike abortive infection, Tai specifically targets phages’ central tail fiber without harming host cells and does not resist the phage by triggering host cell suicide or growth inhibition. He et al. [75] provided evidence that the Tai system and its homologs suppress phage proliferation, functioning as a community-level defense. Furthermore, the phage titer obtained following prophage induction was substantially diminished in the presence of the Tai protein, indicating that Tai differs from superinfection exclusion, as the latter does not influence phage titer.

Another phage tail assembly interference system, known as Tab [76,77], or the “tail assembly blocker,” identified by Patel et al. [76] in *P. aeruginosa* prophage JBD26, mediates a robust anti-phage defense mechanism. Tab expression in *P. aeruginosa* PA14 decreased the plaque formation of five *Casadabanvirus* phages by over 1000-fold. Furthermore, Tab does not degrade the phage genome or inhibit DNA replication. After the prophage is integrated into the host chromosome, Tab’s constitutive expression prepares the cell for defense against further infection. Upon infection by a Tab-sensitive phage, Tab targets the tape measure protein (TMP), inhibiting tail assembly. As the invading phage expresses its late genes like endolysin, the affected cells fail to maintain viability. Tab effectively suppresses the spread of phage infection by blocking active phage progeny release. However, when phage JBD26 enters the lytic cycle, the expression of the late gene operon triggers the production of anti-Tab, which neutralizes Tab’s activity. This mechanism allows JBD26 to efficiently assemble viral particles and successfully complete its life cycle.

Research on Tai and Tab systems highlights the conserved nature of phage structures like tails, making phage assembly interference a simple yet effective means. Both systems, identified within prophages, have corresponding constitutive anti-defense systems. Given the extensive and largely unexplored genetic diversity of temperate phages, prophage-encoded defense and associated anti-defense mechanisms may be a prevalent phenomenon and strategy.

### 2.4. Other Factors Influencing Phage Resistance

#### 2.4.1. Hypoxia and Phage Resistance

Hypoxia may affect phage resistance, as Schumann et al. [78] found that hypoxia reduces the adsorption efficiency of phage PAK_P1 to *P. aeruginosa*, increases resistance frequency, and restricts spontaneous mutation types. Specifically, common spontaneous mutations following phage resistance evolution under both normoxia and hypoxia conditions involved five genes (*amn*, *galU*, *ompH*, *ssg*, and *wapH*), where *galU*, *ssg*, and *wapH* are implicated in LPS biosynthesis (serving as phage-binding receptors) [78]. Mutations induced by different bacterial physiological states under diverse conditions may exhibit specific preferences. Compared with the normoxic condition, hypoxia increases the frequency of *amn* indels and *galU* nSNPs, potentially attributable to stress on large deletions. Additionally, hypoxia may influence phage receptor recognition and adsorption during resistance development [78]. Furthermore, hypoxia can result in decreased cellular motility and metabolic capacity, driving some bacteria into a dormancy state [79,80], which may, consequently, influence phage adsorption and replication.

#### 2.4.2. Biofilm and Phage Resistance

Most studies on phage resistance focus on planktonic bacteria, particularly those cultured under laboratory conditions, where sufficient and direct bacteria–phage interactions enable rapid phage resistance development. In nature, bacterial biofilms prevalently encounter bacteriophages and may influence phage resistance. Biofilms provide bacteria with adaptability to various adverse environments, and the substantial extracellular matrix within biofilms may influence phage resistance. For instance, the CsgA amyloid fiber network within the *E. coli* biofilm inhibits the transport of T7 phage into the biofilm, thereby conferring protection to the bacterial population. Moreover, CsgA fibers also hinder phage attachment by encapsulating cells or directly binding to phage particles [81]. Elhanan Tzipilevich et al. [82] showed that altering potassium efflux from KtrCD channels enhances biofilm formation in *Bacillus subtilis* and thus promotes survival after phage infection.

The known mechanisms of bacterial biofilm resistance to phage infection encompass the following [83]: (i) quorum sensing downregulates the expression of phage receptors, thereby diminishing phage adsorption; (ii) the extracellular matrix restricts phage–bacteria interactions; and (iii) nutrient diffusion limitation creates metabolically dormant regions, inhibiting phage replication. Given the intricate structure and diverse components of biofilms, including those formed by mixed bacterial communities and fungal–bacterial consortia, phage–bacteria interactions are highly complex and multifaceted.

#### 2.4.3. Small Molecules and Phage Resistance

The majority of the anti-phage defenses described so far are mediated by proteins, RNAs, or their complexes. However, bacterial metabolites also play a crucial role, with studies showing that certain bacterial-derived small-molecule metabolites can influence phage resistance. For instance, de Mattos et al. [84] found that bacterial lysate could induce bacteria to develop phage resistance and proved that it was polyamine in the lysates that inhibited phage genome replication through Gac/Rsm signaling in *P. aeruginosa*. In addition, coelichelin, a tripeptide siderophore secreted by *Streptomyces*, can chelate iron to prevent *B. subtilis* from entering the stationary phase and forming spores, thus maintaining its phage-susceptible state and ensuring *Streptomyces*’ competitive advantage [85].

Some bacteria-derived drugs, such as anthracyclines (primarily used in anti-tumor therapy) and aminoglycosides (known for antibacterial effects), may also confer anti-phage defense to resistant strains. For instance, daunorubicin acts at an early stage following phage DNA injection but prior to replication, effectively inhibiting the infection of all tested dsDNA phages [86]. Similarly, aminoglycosides, including kanamycin, hygromycin, and streptomycin, inhibit mycobacterial phage infection by blocking DNA replication [87].

## 3. Emergence and Mechanisms of Phage Resistance in Animal Models

**Figure 3:**
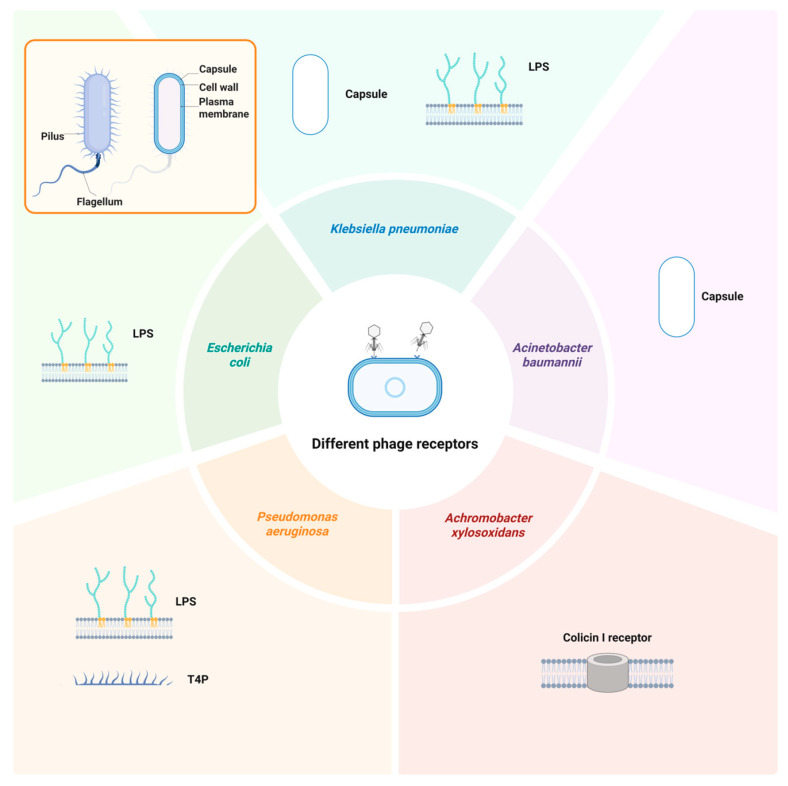
Phage resistance in animal experiments and clinical cases: bacterial species and phage receptors. The five Gram-negative bacterial species and their corresponding phage receptors discussed in this review are color-coded for visual distinction. A schematic diagram illustrating the structure of capsular Gram-negative bacteria is presented in the upper-left panel. Created in BioRender. Shao, Y. (2026) https://BioRender.com/7fplfq2 (accessed on 4 February 2026).

Prior to clinical implementation, the execution of animal experiments constitutes an essential prerequisite. Phage therapy has exhibited remarkable therapeutic efficacy in addressing various bacterial infections during preclinical animal studies [9,88,89,90,91,92]. However, there remain relatively limited studies investigating phage resistance in animal models (Table 1; Figure 3). This is most likely attributed to the fact that bacteria in experimental animals are subjected to dual pressures from both phages and the immune system. Previous studies demonstrated that phages and the immune system exhibit synergistic effects on bacterial elimination [2,5], and acquisition of phage resistance may be accompanied by diminished capacity for bacterial immune evasion [93]. As a result, even if bacteria develop phage resistance, they may still be rapidly eradicated by the immune system. The primary findings concerning phage resistance in animal experiments are summarized as follows:

### 3.1. Phage Resistance in Animal Models of Klebsiella pneumoniae Infection

Fang et al. [9] demonstrated that during the decolonization of carbapenem-resistant *K. pneumoniae* (CRKP) in the mouse gut by phage P24, bacteria developed resistance to the phage. Multiple phage P24-resistant mutants were isolated from mouse feces. Their mutations primarily fell into two categories: (i) an insertion sequence disrupting *wcaJ*, which encodes a glycosyltransferase responsible for initiating the synthesis of colanic acid, the major CPS in Enterobacteriaceae, and (ii) a single-base deletion in *mshA*, located upstream of *wcaJ*, encoding a glycosyltransferase family 4 protein. Both genes are closely associated with capsule formation and virulence in CRKP. These mutations reduced CPS production, thereby inhibiting phage P24 adsorption and resulting in phage resistance.

### 3.2. Phage Resistance in Animal Models of Escherichia coli Infection

Gaborieau et al. [10] examined the phage-resistant mechanism of *E. coli* ExPEC ST127/B2 subgroup strain 536 in response to phage 536_P1 under both in vitro (liquid culture) and in vivo (mouse lung infection model) conditions. They discovered that the phage-resistant mechanism exhibited convergence across both in vitro and in vivo environments, irrespective of external conditions. To be specific, phage resistance occurs either through mechanisms involving phage receptor modification or a capsule-mediated receptor masking. LPS serves as the recognition receptor of phage 536_P1 [10], and its mutation directly results in phage resistance. This result is intuitive and represents a common mutation type widely observed to date. Notably, another type of mutant has thicker capsules than the wild-type strain. The K15 capsule overproduction in mutants creates an effective physical barrier against phage 536_P1. In summary, strain 536 employs at least two strategies to evade predation by phage 536_P1: receptor modification or masking to render it inaccessible. In addition, bacterial clones isolated from the mouse lung infected but not phage-treated group retained their sensitivity to phage 536_P1, indicating that the stress exerted solely by the mouse immune system does not influence the emergence of phage resistance.

### 3.3. Phage Resistance in Animal Models of Pseudomonas aeruginosa Infection

Not entirely consistent with the findings of Gaborieau et al. [10], Ashworth et al. [94] investigated phage resistance using a pulmonary infection mouse model caused by *P. aeruginosa* strain B9 (T2436). The results demonstrate that phage resistance can emerge in vivo in the absence of direct phage interaction. This implies that resistance mechanisms may evolve independently of therapeutic pressure, possibly via host-driven selection or pre-adaptation, thereby posing challenges for phage application in certain niches. Through whole-genome sequencing, the researchers identified a frameshift mutation of the *FC629_24630* gene in mouse lung isolates that were not treated with phage. This gene encodes a glycosyltransferase homologous to PA01 migA, a known rhamnosyltransferase in LPS biosynthesis [95]. In isolates from the phage-treated group, mutations in *FC629_24630* and *FC629_09380* related to LPS biosynthesis were also detected, suggesting LPS modification as a strategy for preventing phage adsorption.

## 4. Emergence and Mechanisms of Phage Resistance in Clinical Settings

With the relentless efforts of scientific researchers, an increasing number of phages are now being utilized in the clinical treatment of drug-resistant bacterial infections. Nevertheless, the number of clinical cases involving phage therapy remains limited, and related research is still insufficient. In particular, studies on phage resistance within the context of clinical phage therapy are exceedingly scarce. The following section outlines several cases of clinical phage resistance (Table 2; Figure 3), aiming to derive valuable insights from these examples.

### 4.1. Phage Resistance in Clinical Case of K. pneumoniae Infection

A 54-year-old male patient was hospitalized after a car accident and subsequently developed a nosocomial multidrug-resistant *K. pneumoniae* pulmonary infection [96]. The patient underwent two sequential treatment regimens: a single nebulized phage (ΦKp_GWPB35) targeting Kp7450, followed by a phage cocktail (ΦKp_GWPB35 + ΦKp_GWPA139) with ongoing antibiotic therapy. With both phage treatment courses completed, the patient’s pulmonary infection showed significant improvement, but sputum culture detected phage-resistant *K. pneumoniae* strains. Comparative genomic analysis revealed that fabF deletion in phage-resistant strains, altering LPS structure and impairing adsorption of phages ΦKp_GWPB35 and ΦKp_GWPA139. In spite of acquiring resistance, the colonization ability and virulence of these strains were significantly diminished.

### 4.2. Phage Resistance in Clinical Case of Achromobacter xylosoxidans Infection

In 2017, a 12-year-old patient with cystic fibrosis experienced airway colonization by pan-resistant *A. xylosoxidans* following lung transplantation. Given the lack of response to multiple antibiotic regimens, phage therapy was administered twice as an alternative treatment [37,97]. Ultimately, the colonization by *A. xylosoxidans* was successfully eradicated. Eight isolates of *A. xylosoxidans* were recovered from bronchoalveolar lavage fluid (BALF) samples, and three of these isolates developed resistance to the original phage. Specifically, two strains (is7R and is8R) harbored missense mutations in the gene encoding colicin I receptor (Cir), which has been validated as a functional phage receptor [98]. The genome of the third resistant strain (is3R) was found to be entirely identical to that of its sensitive counterpart (is1S), suggesting that mechanisms other than genetic mutation, such as epigenetic regulation, may underlie the observed phage resistance.

### 4.3. Phage Resistance in Clinical Cases of P. aeruginosa Infection

Van Nieuwenhuyse et al. [36] reported a case of a young child who developed a pan-drug-resistant *P. aeruginosa* infection following liver transplantation in 2022. The child successfully underwent transplantation, and the infection was subsequently resolved through a combined phage–antibiotic therapy. In this case, the BFC1 phage cocktail (comprising one *S. aureus* phage ISP and two *P. aeruginosa* phages, PNM and 14-1) was selected. During the treatment, four phage-resistant strains were isolated and exhibited genetic alterations in regions associated with the T4P complex. Specifically, *pilB* harbored a missense mutation, while *pilM* was inactivated by IS5 transposase insertion [36,37]. As both PilB and PilM are involved in T4P biosynthesis, and T4P serves as the receptor for *P. aeruginosa* phage PNM [99], resistance to the *P. aeruginosa* phage in this case can be attributed to impaired receptor synthesis.

Similarly, in 2024, Jean-Paul Pirnay et al. [37] documented three additional clinical cases of *P. aeruginosa* infections associated with phage resistance. The infection types in these cases varied—chronic sinusitis, ventilator-associated pneumonia, and pulmonary infection—but all exhibited resistance to the phage PNM, as a cocktail comprising phage PNM and 14-1 was administered in these cases. In high agreement, mutations associated with T4P biogenesis, including SNPs in *pilC* and *pilR* or truncations in *fimV*, were identified in these cases. In conclusion, despite the lack of consistency in the types of *P. aeruginosa* infections across the four cases, all strains developed phage resistance by altering the phage receptor T4P, which may provide some reference for similar strains or similar phage therapy in the future.

In addition to the commonly observed T4P mutations in *P. aeruginosa*, Li et al. [100] recently reported a case of chronic biliary tract infection caused by multidrug-resistant *P. aeruginosa*. The patient’s symptoms initially improved following treatment with a four-phage cocktail. However, from day 5 post-treatment, strains resistant to all therapeutic phages were detected. Large-scale deletions in the genomes of phage-resistant strains W221205 and W221205R caused *waaJ* or *gtaB* mutation, which are involved in O-antigen production. Consequently, the bacteria developed phage resistance due to LPS mutations, which is highly consistent with Ashworth et al.’s in vitro findings [94]. These results suggest that in vitro studies can reliably reflect potential clinical scenarios.

### 4.4. Phage Resistance in Clinical Case of Acinetobacter baumannii Infection

In 2016, a 68-year-old diabetic patient with necrotizing pancreatitis and disseminated multidrug-resistant *A. baumannii* infection was successfully treated with lytic phage [101,102]. Strains resistant to the phage cocktail were isolated within 8 days post-phage treatment. Comparative genomic analysis demonstrated that phage-insensitive strains possessed a mutation in *gtr76* encoding capsular glycosyltransferase. Notably, phage-resistant strains isolated in vitro harbored an identical 6 bp deletion, indicating that bacteria may utilize the same anti-phage mechanism in both in vivo and in vitro settings. Moreover, it substantiates the relevance and predictability of laboratory-based in vitro studies on bacteria–phage interactions to the in vivo context of clinical therapy.

**Table 2:** Emergence and mechanisms of phage resistance in clinical settings.

Species	Strain	Phage	Infection Type	Reasons for Hospitalization	Mechanism	Phage Receptor	References
*Klebsiella pneumoniae*	Kp7450	ΦKp_GWPB35 and ΦKp_GWPA139	Pulmonary infection with multidrug-resistant *K. pneumoniae*	Car accident	Deletion of the *fabF* results in altered phage receptor structure	LPS	[96]
*Achromobacter xylosoxidans*	Is1S	JWAlpha, JWDelta, JWT and 2-1 (APC 1.1 + APC 2.1)	Colonization of the respiratory tract by pan-resistant *A. xylosoxidans*	Lung transplantation	A missense mutation in colicin I receptor Cir	Cir	[37,97]
*Acinetobacter baumannii*	TP1	Maestro and AB-Navy71	Disseminated multidrug-resistant *A. baumannii* infection	Diabetes mellitus with necrotizing pancreatitis	Mutation of the *gtr76* encoding capsular glycosyltransferase	Capsule	[101,102]
*Pseudomonas aeruginosa*	Pa1BS	14-1, PNM and ISP (BFC 1)	Liver transplant infection and bloodstream infection	Liver transplant	Mutations in genes involved in T4P biosynthesis, including *pilB* missense mutation and *pilM* insertion by IS5	T4P	[36,37]
	Unknown	14-1, PNM and ISP (BFC 1)	Chronic sinusitis	Chronic sinusitis	Missense mutation in *pilC* involved in T4P biosynthesis	T4P	[37]
	Unknown	14-1, PNM and PT07	Ventilator-associated pneumonia	Ventilator-associated pneumonia	Missense mutation in *pilR* involved in T4P biosynthesis	T4P	[37]
	Unknown	14-1, PNM and PT07	Pulmonary infection	Pulmonary infection	Truncation of *fimV* impairs T4P biosynthesis	T4P	[37]
	W220606	φPA-A60, φPA-A69, φPA-AP0 and φPA-A78	Chronic biliary tract infection	Complex and recurrent BTI caused by a variety of bacteria	Mutations in *waaJ* or *gtaB* involved in LPS biosynthesis	LPS	[100]

## 5. Conclusions and Outlooks

### 5.1. Anti-Phage Defense Mechanisms Exhibit Greater Diversity In Vitro, Whereas Those In Vivo Appear to Be Less Complex

Through summarizing and analyzing existing reports, it is evident that mechanisms employed by bacteria to combat phage infections in vitro are more diverse, covering most stages of the phage lytic cycle (Figure 1). In contrast, phage-resistant mechanisms in vivo, including animal models and clinical phage therapy, appear to be more limited and predominantly focused on phage receptor abnormalities based on the currently available published evidence (Figure 3). Several factors may account for this discrepancy: (i) In vitro experiments are more extensively conducted due to ease of manipulation and sample accessibility, and clinical observations remain limited. (ii) Reporting bias might play a role. Currently, the literature predominantly documents successful cases, while phage resistance often negatively affects treatment outcomes, leading to underreporting of treatment failures related to phage resistance. (iii) Some anti-phage defense systems are not universally present in all bacteria, with receptor mutation potentially being the most common and versatile defense mechanism. (iv) Receptor mutations may impose lower fitness costs compared with other mechanisms, while fitness trade-offs associated with alternative mechanisms might make it difficult for bacteria to survive in vivo. (v) Many anti-phage defense systems are prophage-related, such as SIE, Tai, and Tab, while lytic phages are predominantly used in treatments. The single resistant mechanisms observed in clinical and animal studies may be linked to this. (vi) Substantial discrepancies may exist between laboratory strains and clinical isolates, which pose challenges in accurately reflecting clinical scenarios. Future research necessitates a broader spectrum of strains that better align with clinical realities.

### 5.2. The Emergence of Phage Resistance Is Frequently Accompanied by Bacterial Fitness Trade-Offs

An increasing number of reports on phage resistance have raised concerns regarding the efficacy of phage therapy. Through the concerted efforts of researchers, it now appears that there is an answer to this question: the development of phage resistance is frequently accompanied by fitness trade-offs, impairing bacterial capabilities such as antibiotic resistance, growth rate, virulence, and immune evasion [93,96,103,104,105,106]. These phenomena occur both in vitro and in vivo, typically through mutations in phage receptors affecting essential molecules for bacterial life activities, such as capsules and efflux pumps. Notably, phage resistance does not always have detrimental effects on bacteria and may enhance resistance to specific antibiotics [93,105]. In general, phage-resistant bacteria often face compromised fitness, increasing their susceptibility to stressors like antibiotics or hosts’ immune responses, thus reducing their pathogenicity and resilience.

### 5.3. Co-Evolution of Phages and Bacteria: Phage Countermeasures

A key distinguishing feature of phage therapy compared with antibiotic therapy is the inherent “living” nature, which enables phages to continuously co-evolve with bacteria during their interactions. The intrinsic predator–prey relationship between phages and bacteria drives bacteria to develop defense mechanisms to safeguard their population. Over prolonged periods of interaction, bacteriophages also employ specific strategies to ensure their own propagation. The currently identified phage counter-defense strategies primarily encompass the following: (i) Phages have developed mutant forms to overcome bacterial resistance, such as the “Appelmans Protocol” [107]. (ii) Some bacterial defense systems are encoded by prophage, which also encode anti-defense mechanisms to avoid autoimmunity, such as TaiEcA (interfering with phage tail assembly) and atiEcA (inhibiting TaiEcA), as well as Tab and anti-Tab mentioned in this review. (iii) Certain phages employ anti-bacterial immune strategies to hinder bacterial defenses, such as phages carrying anti-CRISPR elements that suppress Cas nuclease activity, thereby evading CRISPR-Cas immunity [108,109,110].

### 5.4. Potential Directions of Future Phage Application

Current clinical phage application faces two main challenges. First, individual phages exhibit high specificity but a narrow host range, making it difficult to achieve satisfactory outcomes in cases of mixed bacterial infections. Second, single-phage treatments often develop resistance following administration, leading to suboptimal infection management upon recurrence. However, studies have demonstrated that phage cocktails can effectively broaden the host range, and targeting different receptors can increase the difficulty of developing phage resistance, while also imposing a higher fitness cost [103]. Therefore, future phage applications should prioritize the utilization of phages targeting diverse bacterial structural components. Given that in vitro and in vivo phage resistance evolution may follow a similar trajectory, it might be more efficient to rapidly screen phages effective against phage-resistant bacteria and combine them with the original phages.

In addition to phage cocktails (combinations of distinct phages), numerous alternative combination therapies are projected to assume a pivotal role in the near future. It is not difficult to find that in vivo, in vitro, and in clinical settings, phage-resistant mutations are often accompanied by decreased resistance to certain antibiotics [93,103,104,105,106]. Consequently, the combination of phage therapy and antibiotic treatment holds promise for achieving superior therapeutic outcomes. Likewise, the immune escape capability of phage-resistant bacteria is diminished [93,106], enabling a synergistic approach involving phage and immune system interactions to effectively eradicate bacteria, a strategy that has been validated in relevant studies [2,5]. It is important to highlight that in clinical phage applications, the patient’s immune system may play a critical role and is often accompanied by the concurrent use of multiple antibiotics. Consequently, there may be a synergistic interaction among phages, the immune system, and antibiotics. Furthermore, with the rapid advancement of antibacterial materials in recent years, the integration of phages and antibacterial materials has garnered significant attention. For instance, combining photosensitizers with phages [111,112,113] demonstrates a dual functionality: phages target and lyse bacteria, while antibacterial materials exert potent bactericidal effects. Notably, the entire antibacterial process can even be monitored in real time. Future phage therapy may incorporate this strategy by utilizing more broad-spectrum, potent, and rapid-acting antibacterial agents to effectively control bacterial infections while simultaneously curtailing the emergence of phage resistance.

This work reviews the recent advances in phage resistance across in vitro and in vivo systems, with a detailed discussion of resistance mechanisms identified in clinical phage therapy and animal models. Although phage receptor abnormalities predominate in the available in vivo data, the limited scope of published clinical cases and potential publication bias underscore the need for systematic prospective studies. Our primary objective is to provide some valuable insights for future treatments of clinically refractory infections. Future phage therapy could prioritize the exploration of phage cocktails, combination therapies, and the rapid artificial synthesis or genetic engineering of phages to enhance therapeutic efficacy.

## Figures and Tables

**Table 1:** Emergence and mechanisms of phage resistance in experimental animal models.

Species	Strain	Phage	Models	Mechanism	Receptor	Phenotype	Reference
*Klebsiella pneumoniae*	CRKP strain B0 (015134)	Phage P24	Intestinal *K. pneumoniae* colonization mouse model	Mutations in genes involved in receptor synthesis.	CPS	Mutations in *wcaJ* and *mshA* involved in capsule synthesis, resulting in reduced bacterial CPS, hindered the adsorption of phage P24	[9]
*Escherichia coli*	ExPEC ST127/B2 strain 536	Phage 536_P1	Pulmonary infection mouse model	(i) Mutation in genes related to receptor synthesis; (ii) Receptor masking.	LPS	(i) Mutations in the *waa*, *lpcA*, *rfaE*, and *waaD* associated with LPS synthesis; (ii) The thickening of bacterial capsule shielded the receptor, hindering phage binding	[10]
*Pseudomonas aeruginosa*	B9 (T2436)	Phage PELP20, 4-phage cocktail	Pulmonary infection mouse model	Mutation in genes related to receptor synthesis	LPS	FC629_24630 or FC629_09380 mutation related to LPS biosynthesis	[94]

## Data Availability

No new data were created or analyzed in this study. Data sharing is not applicable to this article.

## Abbreviations

Abbreviations	Academic full name
Abi	abortive infection
*A. baumannii*	*Acinetobacter baumannii*
*A. xylosoxidans*	*Achromobacter xylosoxidans*
BALF	bronchoalveolar lavage fluid
*B. subtilis*	*Bacillus subtilis*
CBASS	cyclic oligonucleotide-based anti-phage signaling systems
Cir	colicin I receptor
CPS	capsular polysaccharides
CRISPR	clustered regularly interspaced short palindromic repeats
CRKP	carbapenem-resistant *Klebsiella pneumoniae*
CWD	cell-wall-deficient
Dcm	DNA cytosine methyltransferase
DNA	deoxyribonucleic acid
dsDNA	double-stranded DNA
DSR2	defense-associated sirtuin 2
*E. coli*	*Escherichia coli*
*K. pneumoniae*	*Klebsiella pneumoniae*
LPS	lipopolysaccharide
*L. monocytogenes*	*Listeria monocytogenes*
msDNA	multi-copy single-stranded DNA
MT	methyltransferase
NAD	Nicotinamide Adenine Dinucleotide
ncRNA	non-coding RNA
nSNP	non-synonymous SNP
OMV	outer membrane vesicle
ORF	open reading frame
pre-crRNA	precursor CRISPR RNA
*P. aeruginosa*	*Pseudomonas aeruginosa*
QS	quorum-sensing
RE	restriction endonuclease
RT	reverse transcriptase
SIE	superinfection exclusion
SIR	sirtuin
SNP	single nucleotide polymorphism
*S. aureus*	*Staphylococcus aureus*
TA	toxin-antitoxin
Tab	tail assembly blocker
Tai	tail assembly inhibition
TMP	tape measure protein
T4P	type IV pilus
*V. alginolyticus*	*Vibrio alginolyticus*

## References

[1] (2024). GBD 2021 Antimicrobial Resistance Collaborators. Global burden of bacterial antimicrobial resistance 1990-2021: A systematic analysis with forecasts to 2050. Lancet.

[2] Abd El-Aziz A.M., Elgaml A., Ali Y.M. (2019). Bacteriophage Therapy Increases Complement-Mediated Lysis of Bacteria and Enhances Bacterial Clearance After Acute Lung Infection With Multidrug-Resistant *Pseudomonas aeruginosa*. J. Infect. Dis..

[3] Agarwal R., Johnson C.T., Imhoff B.R., Donlan R.M., McCarty N.A., Garcia A.J. (2018). Inhaled bacteriophage-loaded polymeric microparticles ameliorate acute lung infections. Nat. Biomed. Eng..

[4] Luo J., Xie L., Liu M., Li Q., Wang P., Luo C. (2022). Bactericidal Synergism between Phage YC#06 and Antibiotics: A Combination Strategy to Target Multidrug-Resistant *Acinetobacter baumannii* in vitro and in vivo. Microbiol. Spectr..

[5] Roach D.R., Leung C.Y., Henry M., Morello E., Singh D., Di Santo J.P., Weitz J.S., Debarbieux L. (2017). Synergy between the Host Immune System and Bacteriophage Is Essential for Successful Phage Therapy against an Acute Respiratory Pathogen. Cell Host Microbe.

[6] Dy R.L., Richter C., Salmond G.P., Fineran P.C. (2014). Remarkable Mechanisms in Microbes to Resist Phage Infections. Annu. Rev. Virol..

[7] Salmond G.P., Fineran P.C. (2015). A century of the phage: Past, present and future. Nat. Rev. Microbiol..

[8] Hyman P. (2017). Phage Receptor☆. Reference Module in Life Sciences.

[9] Fang Q., Feng Y., McNally A., Zong Z. (2022). Characterization of phage resistance and phages capable of intestinal decolonization of carbapenem-resistant *Klebsiella pneumoniae* in mice. Commun. Biol..

[10] Gaborieau B., Delattre R., Adiba S., Clermont O., Denamur E., Ricard J.D., Debarbieux L. (2024). Variable fitness effects of bacteriophage resistance mutations in *Escherichia coli*: Implications for phage therapy. J. Virol..

[11] Chen L., Zhao X., Wongso S., Lin Z., Wang S. (2024). Trade-offs between receptor modification and fitness drive host-bacteriophage co-evolution leading to phage extinction or co-existence. ISME J..

[12] Koderi Valappil S., Shetty P., Deim Z., Terhes G., Urbán E., Váczi S., Patai R., Polgár T., Pertics B.Z., Schneider G. (2021). Survival Comes at a Cost: A Coevolution of Phage and Its Host Leads to Phage Resistance and Antibiotic Sensitivity of *Pseudomonas aeruginosa* Multidrug Resistant Strains. Front. Microbiol..

[13] Rabiey M., Grace E.R., Pawlos P., Bihi M., Ahmed H., Hampson G.E., Al Riyami A., Alharbi L., Sanchez-Lucas R., Korotania N. (2024). Coevolutionary analysis of Pseudomonas syringae-phage interactions to help with rational design of phage treatments. Microb. Biotechnol..

[14] Sumrall E.T., Shen Y., Keller A.P., Rismondo J., Pavlou M., Eugster M.R., Boulos S., Disson O., Thouvenot P., Kilcher S. (2019). Phage resistance at the cost of virulence: Listeria monocytogenes serovar 4b requires galactosylated teichoic acids for InlB-mediated invasion. PLoS Pathog..

[15] Sumrall E.T., Schneider S.R., Boulos S., Loessner M.J., Shen Y. (2021). Glucose Decoration on Wall Teichoic Acid Is Required for Phage Adsorption and InlB-Mediated Virulence in *Listeria ivanovii*. J. Bacteriol..

[16] Holst Sørensen M.C., van Alphen L.B., Fodor C., Crowley S.M., Christensen B.B., Szymanski C.M., Brøndsted L. (2012). Phase variable expression of capsular polysaccharide modifications allows *Campylobacter jejuni* to avoid bacteriophage infection in chickens. Front. Cell. Infect. Microbiol..

[17] Høyland-Kroghsbo N.M., Maerkedahl R.B., Svenningsen S.L. (2013). A quorum-sensing-induced bacteriophage defense mechanism. mBio.

[18] Li X., Zhang C., Li S., Liang S., Xu X., Zhao Z. (2024). Quorum sensing positively regulates CPS-dependent Autographiviridae phage infection in Vibrio alginolyticus. Appl. Environ. Microbiol..

[19] Broniewski J.M., Chisnall M.A.W., Høyland-Kroghsbo N.M., Buckling A., Westra E.R. (2021). The effect of Quorum sensing inhibitors on the evolution of CRISPR-based phage immunity in *Pseudomonas aeruginosa*. ISME J..

[20] Wohlfarth J.C., Feldmüller M., Schneller A., Kilcher S., Burkolter M., Meile S., Pilhofer M., Schuppler M., Loessner M.J. (2023). L-form conversion in Gram-positive bacteria enables escape from phage infection. Nat. Microbiol..

[21] Ongenae V., Mabrouk A.S., Crooijmans M., Rozen D., Briegel A., Claessen D. (2022). Reversible bacteriophage resistance by shedding the bacterial cell wall. Open Biol..

[22] Destoumieux-Garzón D., Duquesne S., Peduzzi J., Goulard C., Desmadril M., Letellier L., Rebuffat S., Boulanger P. (2005). The iron-siderophore transporter FhuA is the receptor for the antimicrobial peptide microcin J25: Role of the microcin Val11-Pro16 beta-hairpin region in the recognition mechanism. Biochem. J..

[23] Augustyniak D., Olszak T., Drulis-Kawa Z. (2022). Outer Membrane Vesicles (OMVs) of *Pseudomonas aeruginosa* Provide Passive Resistance but Not Sensitization to LPS-Specific Phages. Viruses.

[24] Manning A.J., Kuehn M.J. (2011). Contribution of bacterial outer membrane vesicles to innate bacterial defense. BMC Microbiol..

[25] Reyes-Robles T., Dillard R.S., Cairns L.S., Silva-Valenzuela C.A., Housman M., Ali A., Wright E.R., Camilli A. (2018). Vibrio cholerae Outer Membrane Vesicles Inhibit Bacteriophage Infection. J. Bacteriol..

[26] Stephan M.S., Broeker N.K., Saragliadis A., Roos N., Linke D., Barbirz S. (2020). In vitro Analysis of O-Antigen-Specific Bacteriophage P22 Inactivation by Salmonella Outer Membrane Vesicles. Front. Microbiol..

[27] Leavitt J.C., Woodbury B.M., Gilcrease E.B., Bridges C.M., Teschke C.M., Casjens S.R. (2024). Bacteriophage P22 SieA-mediated superinfection exclusion. mBio.

[28] Chung I.Y., Jang H.J., Bae H.W., Cho Y.H. (2014). A phage protein that inhibits the bacterial ATPase required for type IV pilus assembly. Proc. Natl. Acad. Sci. USA.

[29] Schmidt A.K., Fitzpatrick A.D., Schwartzkopf C.M., Faith D.R., Jennings L.K., Coluccio A., Hunt D.J., Michaels L.A., Hargil A., Chen Q. (2022). A Filamentous Bacteriophage Protein Inhibits Type IV Pili To Prevent Superinfection of *Pseudomonas aeruginosa*. mBio.

[30] Newton G.J., Daniels C., Burrows L.L., Kropinski A.M., Clarke A.J., Lam J.S. (2001). Three-component-mediated serotype conversion in *Pseudomonas aeruginosa* by bacteriophage D3. Mol. Microbiol..

[31] Shah M., Taylor V.L., Bona D., Tsao Y., Stanley S.Y., Pimentel-Elardo S.M., McCallum M., Bondy-Denomy J., Howell P.L., Nodwell J.R. (2021). A phage-encoded anti-activator inhibits quorum sensing in *Pseudomonas aeruginosa*. Mol. Cell.

[32] Ko C.C., Hatfull G.F. (2018). Mycobacteriophage Fruitloop gp52 inactivates Wag31 (DivIVA) to prevent heterotypic superinfection. Mol. Microbiol..

[33] Cumby N., Edwards A.M., Davidson A.R., Maxwell K.L. (2012). The bacteriophage HK97 gp15 moron element encodes a novel superinfection exclusion protein. J. Bacteriol..

[34] Cumby N., Reimer K., Mengin-Lecreulx D., Davidson A.R., Maxwell K.L. (2015). The phage tail tape measure protein, an inner membrane protein and a periplasmic chaperone play connected roles in the genome injection process of *E. coli* phage HK97. Mol. Microbiol..

[35] Castang S., Dove S.L. (2012). Basis for the essentiality of H-NS family members in *Pseudomonas aeruginosa*. J. Bacteriol..

[36] Van Nieuwenhuyse B., Van der Linden D., Chatzis O., Lood C., Wagemans J., Lavigne R., Schroven K., Paeshuyse J., de Magnée C., Sokal E. (2022). Bacteriophage-antibiotic combination therapy against extensively drug-resistant *Pseudomonas aeruginosa* infection to allow liver transplantation in a toddler. Nat. Commun..

[37] Pirnay J.-P., Djebara S., Steurs G., Griselain J., Cochez C., De Soir S., Glonti T., Spiessens A., Vanden Berghe E., Green S. (2024). Personalized bacteriophage therapy outcomes for 100 consecutive cases: A multicentre, multinational, retrospective observational study. Nat. Microbiol..

[38] Barrangou R., Fremaux C., Deveau H., Richards M., Boyaval P., Moineau S., Romero D.A., Horvath P. (2007). CRISPR provides acquired resistance against viruses in prokaryotes. Science.

[39] Amitai G., Sorek R. (2016). CRISPR-Cas adaptation: Insights into the mechanism of action. Nat. Rev. Microbiol..

[40] van Houte S., Buckling A., Westra E.R. (2016). Evolutionary Ecology of Prokaryotic Immune Mechanisms. Microbiol. Mol. Biol. Rev..

[41] Xiong X., Wu G., Wei Y., Liu L., Zhang Y., Su R., Jiang X., Li M., Gao H., Tian X. (2020). SspABCD-SspE is a phosphorothioation-sensing bacterial defence system with broad anti-phage activities. Nat. Microbiol..

[42] Wang S., Wan M., Huang R., Zhang Y., Xie Y., Wei Y., Ahmad M., Wu D., Hong Y., Deng Z. (2021). SspABCD-SspFGH Constitutes a New Type of DNA Phosphorothioate-Based Bacterial Defense System. mBio.

[43] Wang S., Sun E., Liu Y., Yin B., Zhang X., Li M., Huang Q., Tan C., Qian P., Rao V.B. (2023). Landscape of New Nuclease-Containing Antiphage Systems in *Escherichia coli* and the Counterdefense Roles of Bacteriophage T4 Genome Modifications. J. Virol..

[44] Vassallo C.N., Doering C.R., Littlehale M.L., Teodoro G.I.C., Laub M.T. (2022). A functional selection reveals previously undetected anti-phage defence systems in the *E. coli* pangenome. Nat. Microbiol..

[45] Bravo J.P.K., Aparicio-Maldonado C., Nobrega F.L., Brouns S.J.J., Taylor D.W. (2022). Structural basis for broad anti-phage immunity by DISARM. Nat. Commun..

[46] Goldfarb T., Sberro H., Weinstock E., Cohen O., Doron S., Charpak-Amikam Y., Afik S., Ofir G., Sorek R. (2015). BREX is a novel phage resistance system widespread in microbial genomes. EMBO J..

[47] Tesson F., Hervé A., Mordret E., Touchon M., D’humières C., Cury J., Bernheim A. (2022). Systematic and quantitative view of the antiviral arsenal of prokaryotes. Nat. Commun..

[48] Drobiazko A., Adams M.C., Skutel M., Potekhina K., Kotovskaya O., Trofimova A., Matlashov M., Yatselenko D., Maxwell K.L., Blower T.R. (2025). Molecular basis of foreign DNA recognition by BREX anti-phage immunity system. Nat. Commun..

[49] Lopatina A., Tal N., Sorek R. (2020). Abortive Infection. Bacterial Suicide as an Antiviral Immune Strategy. Annu. Rev. Virol..

[50] Rostøl J.T., Marraffini L. (2019). (Ph)ighting Phages: How Bacteria Resist Their Parasites. Cell Host Microbe.

[51] Snyder L. (1995). Phage-exclusion enzymes: A bonanza of biochemical and cell biology reagents?. Mol. Microbiol..

[52] Parma D.H., Snyder M., Sobolevski S., Nawroz M., Brody E., Gold L. (1992). The Rex system of bacteriophage lambda: Tolerance and altruistic cell death. Genes Dev..

[53] Durmaz E., Klaenhammer T.R. (2007). Abortive phage resistance mechanism AbiZ speeds the lysis clock to cause premature lysis of phage-infected *Lactococcus lactis*. J. Bacteriol..

[54] Guo Y., Quiroga C., Chen Q., McAnulty M.J., Benedik M.J., Wood T.K., Wang X. (2014). RalR (a DNase) and RalA (a small RNA) form a type I toxin-antitoxin system in *Escherichia coli*. Nucleic Acids Res..

[55] Mets T., Lippus M., Schryer D., Liiv A., Kasari V., Paier A., Maiväli Ü., Remme J., Tenson T., Kaldalu N. (2017). Toxins MazF and MqsR cleave *Escherichia coli* rRNA precursors at multiple sites. RNA Biol..

[56] Weel-Sneve R., Kristiansen K.I., Odsbu I., Dalhus B., Booth J., Rognes T., Skarstad K., Bjørås M. (2013). Single transmembrane peptide DinQ modulates membrane-dependent activities. PLoS Genet..

[57] Wang Z., Chen W., Wang E., Le S., Han W., Yuan S., Gu J., Liu B. (2025). Battle beyond membrane: Flagella as a conduit for phage DNA entry and a trigger for bacterial defense in *Yersinia enterocolitica*. Nucleic Acids Res..

[58] Cohen D., Melamed S., Millman A., Shulman G., Oppenheimer-Shaanan Y., Kacen A., Doron S., Amitai G., Sorek R. (2019). Cyclic GMP-AMP signalling protects bacteria against viral infection. Nature.

[59] Whiteley A.T., Eaglesham J.B., de Oliveira Mann C.C., Morehouse B.R., Lowey B., Nieminen E.A., Danilchanka O., King D.S., Lee A.S.Y., Mekalanos J.J. (2019). Bacterial cGAS-like enzymes synthesize diverse nucleotide signals. Nature.

[60] Zeng Z., Hu Z., Zhao R., Rao J., Mestre M.R., Liu Y., Liu S., Feng H., Chen Y., He H. (2025). Base-modified nucleotides mediate immune signaling in bacteria. Science.

[61] Millman A., Bernheim A., Stokar-Avihail A., Fedorenko T., Voichek M., Leavitt A., Oppenheimer-Shaanan Y., Sorek R. (2020). Bacterial Retrons Function In Anti-Phage Defense. Cell.

[62] Wang Y., Guan Z., Wang C., Nie Y., Chen Y., Qian Z., Cui Y., Xu H., Wang Q., Zhao F. (2022). Cryo-EM structures of *Escherichia coli* Ec86 retron complexes reveal architecture and defence mechanism. Nat. Microbiol..

[63] Gao L., Altae-Tran H., Böhning F., Makarova K.S., Segel M., Schmid-Burgk J.L., Koob J., Wolf Y.I., Koonin E.V., Zhang F. (2020). Diverse enzymatic activities mediate antiviral immunity in prokaryotes. Science.

[64] Wang C., Rish A.D., Armbruster E.G., Xie J., Loveland A.B., Shen Z., Gu B., Korostelev A.A., Pogliano J., Fu T.M. (2025). Disassembly activates Retron-Septu for antiphage defense. Science.

[65] Dillingham M.S., Kowalczykowski S.C. (2008). RecBCD enzyme and the repair of double-stranded DNA breaks. Microbiol. Mol. Biol. Rev..

[66] Wang Y., Wang C., Guan Z., Cao J., Xu J., Wang S., Cui Y., Wang Q., Chen Y., Yin Y. (2024). DNA methylation activates retron Ec86 filaments for antiphage defense. Cell Rep..

[67] Tuck O.T., Adler B.A., Armbruster E.G., Lahiri A., Hu J.J., Zhou J., Pogliano J., Doudna J.A. (2024). Genome integrity sensing by the broad-spectrum Hachiman antiphage defense complex. Cell.

[68] Doron S., Melamed S., Ofir G., Leavitt A., Lopatina A., Keren M., Amitai G., Sorek R. (2018). Systematic discovery of antiphage defense systems in the microbial pangenome. Science.

[69] Hu H., Popp P.F., Hughes T.C.D., Roa-Eguiara A., Rutbeek N.R., Martin F.J.O., Hendriks I.A., Payne L.J., Yan Y., Humolli D. (2024). Structure and mechanism of the Zorya anti-phage defence system. Nature.

[70] Zhen X., Zhou B., Liu Z., Wang X., Zhao H., Wu S., Li Z., Liang J., Zhang W., Zhu Q. (2024). Mechanistic basis for the allosteric activation of NADase activity in the Sir2-HerA antiphage defense system. Nat. Commun..

[71] Zaremba M., Dakineviciene D., Golovinas E., Zagorskaitė E., Stankunas E., Lopatina A., Sorek R., Manakova E., Ruksenaite A., Silanskas A. (2022). Short prokaryotic Argonautes provide defence against incoming mobile genetic elements through NAD(+) depletion. Nat. Microbiol..

[72] Wang X., Li X., Yu G., Zhang L., Zhang C., Wang Y., Liao F., Wen Y., Yin H., Liu X. (2023). Structural insights into mechanisms of Argonaute protein-associated NADase activation in bacterial immunity. Cell Res..

[73] Ka D., Oh H., Park E., Kim J.H., Bae E. (2020). Structural and functional evidence of bacterial antiphage protection by Thoeris defense system via NAD(+) degradation. Nat. Commun..

[74] Yin H., Li X., Wang X., Zhang C., Gao J., Yu G., He Q., Yang J., Liu X., Wei Y. (2024). Insights into the modulation of bacterial NADase activity by phage proteins. Nat. Commun..

[75] He L., Miguel-Romero L., Patkowski J.B., Alqurainy N., Rocha E.P.C., Costa T.R.D., Fillol-Salom A., Penadés J.R. (2024). Tail assembly interference is a common strategy in bacterial antiviral defenses. Nat. Commun..

[76] Patel P.H., Taylor V.L., Zhang C., Getz L.J., Fitzpatrick A.D., Davidson A.R., Maxwell K.L. (2024). Anti-phage defence through inhibition of virion assembly. Nat. Commun..

[77] Tsao Y.F., Taylor V.L., Kala S., Bondy-Denomy J., Khan A.N., Bona D., Cattoir V., Lory S., Davidson A.R., Maxwell K.L. (2018). Phage Morons Play an Important Role in *Pseudomonas aeruginosa* Phenotypes. J. Bacteriol..

[78] Schumann A.R., Sue A.D., Roach D.R. (2022). Hypoxia Increases the Tempo of Phage Resistance and Mutational Bottlenecking of *Pseudomonas aeruginosa*. Front. Microbiol..

[79] Schubert O.T., Ludwig C., Kogadeeva M., Zimmermann M., Rosenberger G., Gengenbacher M., Gillet L.C., Collins B.C., Röst H.L., Kaufmann S.H. (2015). Absolute Proteome Composition and Dynamics during Dormancy and Resuscitation of *Mycobacterium tuberculosis*. Cell Host Microbe.

[80] Kundu M., Basu J. (2021). Applications of Transcriptomics and Proteomics for Understanding Dormancy and Resuscitation in *Mycobacterium tuberculosis*. Front. Microbiol..

[81] Vidakovic L., Singh P.K., Hartmann R., Nadell C.D., Drescher K. (2017). Dynamic biofilm architecture confers individual and collective mechanisms of viral protection. Nat. Microbiol..

[82] Tzipilevich E., Benfey P.N. (2021). Phage-Resistant Bacteria Reveal a Role for Potassium in Root Colonization. mBio.

[83] Pires D.P., Melo L.D.R., Azeredo J. (2021). Understanding the Complex Phage-Host Interactions in Biofilm Communities. Annu. Rev. Virol..

[84] de Mattos C.D., Faith D.R., Nemudryi A.A., Schmidt A.K., Bublitz D.C., Hammond L., Kinnersley M.A., Schwartzkopf C.M., Robinson A.J., Joyce A. (2023). Polyamines and linear DNA mediate bacterial threat assessment of bacteriophage infection. Proc. Natl. Acad. Sci. USA.

[85] Zang Z., Zhang C., Park K.J., Schwartz D.A., Podicheti R., Lennon J.T., Gerdt J.P. (2025). Streptomyces secretes a siderophore that sensitizes competitor bacteria to phage infection. Nat. Microbiol..

[86] Kronheim S., Daniel-Ivad M., Duan Z., Hwang S., Wong A.I., Mantel I., Nodwell J.R., Maxwell K.L. (2018). A chemical defence against phage infection. Nature.

[87] Jiang Z., Wei J., Liang Y., Peng N., Li Y. (2020). Aminoglycoside Antibiotics Inhibit Mycobacteriophage Infection. Antibiotics.

[88] Anand T., Virmani N., Kumar S., Mohanty A.K., Pavulraj S., Bera B.C., Vaid R.K., Ahlawat U., Tripathi B.N. (2020). Phage therapy for treatment of virulent *Klebsiella pneumoniae* infection in a mouse model. J. Glob. Antimicrob. Resist..

[89] Zhang Y., Shao Y., You H., Shen Y., Miao F., Yuan C., Chen X., Zhai M., Shen Y., Zhang J. (2024). Characterization and therapeutic potential of MRABP9, a novel lytic bacteriophage infecting multidrug-resistant *Acinetobacter baumannii* clinical strains. Virology.

[90] Yin S., Huang G., Zhang Y., Jiang B., Yang Z., Dong Z., You B., Yuan Z., Hu F., Zhao Y. (2017). Phage Abp1 Rescues Human Cells and Mice from Infection by Pan-Drug Resistant *Acinetobacter baumannii*. Cell. Physiol. Biochem..

[91] Alemayehu D., Casey P.G., McAuliffe O., Guinane C.M., Martin J.G., Shanahan F., Coffey A., Ross R.P., Hill C. (2012). Bacteriophages φMR299-2 and φNH-4 can eliminate *Pseudomonas aeruginosa* in the murine lung and on cystic fibrosis lung airway cells. mBio.

[92] Hesse S., Malachowa N., Porter A.R., Freedman B., Kobayashi S.D., Gardner D.J., Scott D.P., Adhya S., DeLeo F.R. (2021). Bacteriophage Treatment Rescues Mice Infected with Multidrug-Resistant *Klebsiella pneumoniae* ST258. mBio.

[93] Gordillo Altamirano F., Forsyth J.H., Patwa R., Kostoulias X., Trim M., Subedi D., Archer S.K., Morris F.C., Oliveira C., Kielty L. (2021). Bacteriophage-resistant *Acinetobacter baumannii* are resensitized to antimicrobials. Nat. Microbiol..

[94] Ashworth E.A., Wright R.C.T., Shears R.K., Wong J.K.L., Hassan A., Hall J.P.J., Kadioglu A., Fothergill J.L. (2024). Exploiting lung adaptation and phage steering to clear pan-resistant *Pseudomonas aeruginosa* infections in vivo. Nat. Commun..

[95] Poon K.K., Westman E.L., Vinogradov E., Jin S., Lam J.S. (2008). Functional characterization of MigA and WapR: Putative rhamnosyltransferases involved in outer core oligosaccharide biosynthesis of *Pseudomonas aeruginosa*. J. Bacteriol..

[96] Li J., Yan B., He B., Li L., Zhou X., Wu N., Wang Q., Guo X., Zhu T., Qin J. (2023). Development of phage resistance in multidrug-resistant *Klebsiella pneumoniae* is associated with reduced virulence: A case report of a personalised phage therapy. Clin. Microbiol. Infect..

[97] Lebeaux D., Merabishvili M., Caudron E., Lannoy D., Van Simaey L., Duyvejonck H., Guillemain R., Thumerelle C., Podglajen I., Compain F. (2021). A Case of Phage Therapy against Pandrug-Resistant *Achromobacter xylosoxidans* in a 12-Year-Old Lung-Transplanted Cystic Fibrosis Patient. Viruses.

[98] Hantke K. (2020). Compilation of *Escherichia coli* K-12 outer membrane phage receptors—Their function and some historical remarks. FEMS Microbiol. Lett..

[99] Ceyssens P.J., Glonti T., Kropinski N.M., Lavigne R., Chanishvili N., Kulakov L., Lashkhi N., Tediashvili M., Merabishvili M. (2011). Phenotypic and genotypic variations within a single bacteriophage species. Virol. J..

[100] Li N., Li L., He B., Li D., Jin W., Wu Y., Zhu B., Cheng M., Wu N., Tan D. (2025). Personalized bacteriophage therapy for chronic biliary tract *Pseudomonas aeruginosa* infections. hLife.

[101] Liu M., Hernandez-Morales A., Clark J., Le T., Biswas B., Bishop-Lilly K.A., Henry M., Quinones J., Voegtly L.J., Cer R.Z. (2022). Comparative genomics of *Acinetobacter baumannii* and therapeutic bacteriophages from a patient undergoing phage therapy. Nat. Commun..

[102] Schooley R.T., Biswas B., Gill J.J., Hernandez-Morales A., Lancaster J., Lessor L., Barr J.J., Reed S.L., Rohwer F., Benler S. (2017). Development and Use of Personalized Bacteriophage-Based Therapeutic Cocktails To Treat a Patient with a Disseminated Resistant *Acinetobacter baumannii* Infection. Antimicrob. Agents Chemother..

[103] Gao D., Ji H., Wang L., Li X., Hu D., Zhao J., Wang S., Tao P., Li X., Qian P. (2022). Fitness Trade-Offs in Phage Cocktail-Resistant Salmonella enterica Serovar Enteritidis Results in Increased Antibiotic Susceptibility and Reduced Virulence. Microbiol. Spectr..

[104] Burmeister A.R., Fortier A., Roush C., Lessing A.J., Bender R.G., Barahman R., Grant R., Chan B.K., Turner P.E. (2020). Pleiotropy complicates a trade-off between phage resistance and antibiotic resistance. Proc. Natl. Acad. Sci. USA.

[105] Li N., Zeng Y., Wang M., Bao R., Chen Y., Li X., Pan J., Zhu T., Hu B., Tan D. (2022). Characterization of Phage Resistance and Their Impacts on Bacterial Fitness in *Pseudomonas aeruginosa*. Microbiol. Spectr..

[106] Rotman E., McClure S., Glazier J., Fuerte-Stone J., Foldi J., Erani A., McGann R., Arnold J., Lin H., Valaitis S. (2024). Rapid design of bacteriophage cocktails to suppress the burden and virulence of gut-resident carbapenem-resistant *Klebsiella pneumoniae*. Cell Host Microbe.

[107] Burrowes B.H., Molineux I.J., Fralick J.A. (2019). Directed in vitro Evolution of Therapeutic Bacteriophages: The Appelmans Protocol. Viruses.

[108] Stanley S.Y., Maxwell K.L. (2018). Phage-Encoded Anti-CRISPR Defenses. Annu. Rev. Genet..

[109] Bondy-Denomy J., Pawluk A., Maxwell K.L., Davidson A.R. (2013). Bacteriophage genes that inactivate the CRISPR/Cas bacterial immune system. Nature.

[110] Camara-Wilpert S., Mayo-Muñoz D., Russel J., Fagerlund R.D., Madsen J.S., Fineran P.C., Sørensen S.J., Pinilla-Redondo R. (2023). Bacteriophages suppress CRISPR-Cas immunity using RNA-based anti-CRISPRs. Nature.

[111] Ran B., Yuan Y., Xia W., Li M., Yao Q., Wang Z., Wang L., Li X., Xu Y., Peng X. (2020). A photo-sensitizable phage for multidrug-resistant *Acinetobacter baumannii* therapy and biofilm ablation. Chem. Sci..

[112] Wang L., Fan X., Gonzalez Moreno M., Tkhilaishvili T., Du W., Zhang X., Nie C., Trampuz A., Haag R. (2022). Photocatalytic Quantum Dot-Armed Bacteriophage for Combating Drug-Resistant Bacterial Infection. Adv. Sci..

[113] He X., Yang Y., Guo Y., Lu S., Du Y., Li J.J., Zhang X., Leung N.L.C., Zhao Z., Niu G. (2020). Phage-Guided Targeting, Discriminative Imaging, and Synergistic Killing of Bacteria by AIE Bioconjugates. J. Am. Chem. Soc..

